# Knockdown of DEPDC1B inhibits the development of glioblastoma

**DOI:** 10.1186/s12935-020-01404-7

**Published:** 2020-07-15

**Authors:** Xu Chen, Zheng-Qian Guo, Dan Cao, Yong Chen, Jian Chen

**Affiliations:** Department of Neurosurgery, Tongji Hospital, Tongji Medical College, Huazhong University of Science and Technology, Jiefang Ave, 1095, Wuhan, 430030 China

**Keywords:** GBM, DEPDC1B, Proliferation, Apoptosis, Migration

## Abstract

**Background:**

Glioblastoma (GBM) is the most common primary malignant brain tumor in adults with a poor prognosis. DEPDC1B (DEP domain-containing protein 1B) has been shown to be associated with some types of malignancies. However, the role and underlying regulatory mechanisms of DEPDC1B in GBM remain elusive.

**Methods:**

In this research, the expression level of DEPDC1B in GBM tissues was detected by IHC. The DEPDC1B knockdown cell line was constructed, identified by qRT-PCR and western blot and used to construct the xenotransplantation mice model and intracranial xenograft model. MTT assay, colony formation assay, flow cytometry, and Transwell assay were used to detected cell proliferation, apoptosis and migration.

**Results:**

The results proved that DEPDC1B was significantly upregulated in tumor tissues, and silencing DEPDC1B could inhibit proliferation, migration and promote apoptosis of GBM cell. In addition, human apoptosis antibody array detection showed that after DEPDC1B knockdown, the expression of apoptosis-related proteins was downregulated, such as IGFBP-2, Survivin, N-cadherin, Vimentin and Snail. Finally, we indicated that knockdown of DEPDC1B significantly inhibited tumor growth in vivo.

**Conclusions:**

In summary, DEPDC1B was involved in the development and progression of GBM, which may be a potential therapeutic target and bring a breakthrough in the treatment.

## Introduction

Glioblastoma multiforme (GBM) is a lethal malignancy of the central nervous system (CNS) [1], accounting for approximately 15% of all primary brain tumors and 60% of all astrocytomas [2]. GBM mainly originates from low-grade astrocytoma and has been classified as grade IV astrocytoma by the world health organization [2]. At present, the treatment of GBM is mainly tumor resection, followed by adjuvant radiotherapy and temozolomide [3]. Although this standardized treatment has shown effectiveness in extending patient survival, the prognosis is still extremely poor, with a median survival (MS) of 14.6 months and an average 5-year survival of less than 5% [1, 4, 5]. Part of the reason may be the ability of GBM cells to spread and invade into the surrounding brain parenchyma and their resistance to treatment [6, 7]. Therefore, understanding the mechanisms that cause the disease to progress is essential for developing more effective therapy.

DEP domain-containing protein 1B (DEPDC1B) was located on chromosome 5 (5q12.1), which encodes DEPDC1B protein and containing two conserved domains, DEP domain and RhoGAP domain [8–10]. The DEP domain is a spherical domain containing about 90 amino acids, which was first identified and named in three proteins: drosophila, Caenorhabditis elegans EGL-10 and mammalian Pleckstrin [11, 12]. DEP, which enables the protein to interact with the G protein coupled receptors as well as negatively charged membrane phospholipids, which is necessary for WNT signaling [9]. RhoGAP is responsible for Rho GTPase signaling [13]. It is speculated that the expression regulation is positively regulated by P53, which is supported by the fact that P63 binding site exists at DEPDC1B transcription initiation site 27 kb [14]. However, the mechanism remains unclear. The interaction between DEPDC1B mediated cell cycle progression and desorption events during mitotic entry has been identified at an early stage [15]. In recent years, it has been reported that DEPDC1B is involved in the regulation of cell activities, including cell growth, movement, differentiation, cell cycle and reactive oxygen species [10]. However, the precise function of DEPDC1B is uncharacterized and its role in GBM is also still unclear.

## Materials and methods

### Immunohistochemical staining (IHC)

Formalin fixed paraffin-embedded (FFPE) tissues were purchased from Shanghai Outdo Biotech Company, which included 180 GBM tissues and matched normal tissues. The inclusion criteria of the FFPE GBM samples included in this study were the samples of patients with GBM for survival period. Patients with carcinoma in situ (with or without micro invasion) and inflammatory GBM were excluded. FFPE tissues were blind-checked by three pathologists for the pathological details. Xylene were used for paraffin section dewaxing 15 min per time and 100% alcohol for hydration 10 min. After repairing and blocking of the citrate antigen, the sample and DEPDC1B antibody (1: 100, Abcam, USA, # ab124182) were incubated overnight in an incubator at 4 °C. After elution with PBS for five times, secondary antibody IgG (1: 400, Abcam, USA, # ab6721) was added, incubated at room temperature for 30 min, and washed with PBS for three times. Tissue slices were first stained with DAB, and then with hematoxylin. Images were collected with a photomicroscope and analyzed. Finally, the high and moderate expression parameters were determined by the median of IHC experimental scores of all tissues.

### Cell culture

GBM cell lines U87 and U251 were procured from cell bank of the Shanghai Academy of Sciences in China. The cells were cultured in DMEM medium (Gibco, Carlsbad, CA, USA) in 37 °C, 5% CO_2_ atmosphere and supplemented with 10% fetal bovine serum (FBS, Life Technologies, USA) and 1% penicillin/streptomycin.

### Target gene RNA interference by lentiviral vector

Two RNA interference target sequences (sh DEPDC1B-1: 5′-GCTGCTAGATTGGTAACGTTT-3′; sh DEPDC1B-2: 5′-ACAAGCGTCACAGTATTGCAA-3′) were designed with DEPDC1B as template, and two target gene RNA interference lentivirus vectors were constructed, respectively. Firstly, BR-V112 linearized vector was obtained by enzyme digestion of Age I (NEB, Cat. # R3101L) and EcoR I (NEB, Cat. # R3101L). T4 DNA ligase (Thermo Scientific, Cat. # EL0016) was used to connect the linearized vector with annealed double-stranded DNA. Monoclonal on the plate was selected for PCR identification, and the positive clones were sequenced and analyzed. The clones were cultured and extracted to obtain high purity plasmid (EndoFree midi Plasmid Kit, TIANGEN, Beijing, China, # DP117). 293T cells were transfected with three plasmids (tool vector plasmid BR-V112 carrying target sequence, Helper 1.0 and Helper 2.0 for virus packaging), and the transfection agent was Yiberry transfection reagent (Yiberry, Shanghai, China). Lentiviral harvest was performed 48–72 h after transfection. Followed by concentration and purification of lentivirus preservation solution with high titer according to experimental requirements, and the quality standard of the transfected lentivirus was tested again. Then, the physical, sterile, and viral titer status of lentivirus is detected after it can be used for downstream infection efficiency detection. Lentivirus vectors were labeled with fluorescence and observed under fluorescence microscopy 72–96 h after transfection (GFP, Cherry). If the fluorescence efficiency of the cells was more than 80%, the transfection experiment was successful.

### qRT-PCR

Total RNA was extracted from cell samples according to the kit instructions of Trizol reagent (Invitrogen, Carlsbad, CA, USA). The cDNA was obtained by reverse transcription using the Promega M-MLV kit. The qRT-PCR was performed by using AceQ qPCR SYBR Green Master Mix (Vazyme, Nanjing, China). GAPDH was used as a reference control.

**Table:** 

Primer	Sequence
DEPDC1B Primer-F	CTGAAGTGACCCGCAAACAAA
DEPDC1B Primer-R	CTGGTGGGAGATCATTCCATTC
GAPDH Primer-F	TGACTTCAACAGCGACACCCA
GAPDH Primer-R	CACCCTGTTGCTGTAGCCAAA

### Western blot analysis

U87 and U251 cell were seed at the 24-well plate (2 mL/well) at the density of 80,000 cell/well until cell confluency degree reached 85%, and lysed with 1× Lysis Buffer (Cell Signal Technology, Danvers, MA, USA). Quantitative extraction of proteins detected with BCA Protein Assay Kit (HyClone-Pierce, Waltham, MA, USA, # 23225). Then western blot analysis was performed by SDS-PAGE (10%). The protein was transferred to polyvinylidene fluoride (PVDF) membrane using a transfer electrophoresis device at 4 °C and 300 mA constant current for 150 min. Sealing PVDF membrane with sealant (TBST solution with 5% skimmed milk powder) at room temperature for 1 h, then incubated overnight at 4 °C with the following primary antibodies (see primary antibody information table for western blot) and GAPDH antibody. After washing with TBST, the blot was incubated with horseradish peroxidase (HRP) labeled polyclonal secondary antibody (1:3000) (Beyotime, Beijing, China, # A0208) at room temperature for 1 h. Finally, ECL and plus TM western blot system kit (Amersham, Chalfont, UK, # RPN2232) were used for color development.

**Table:** 

Name of antibody	Protein size (kDa)	Diluted multiples	Source of antibody	Company	Number
DEPDC1B	62/70	1:1000	Rabbit	Abcam	ab124182
N-cadherin	125	1:1000	Rabbit	Abcam	ab18203
Vimentin	54	1:2000	Rabbit	Abcam	ab92547
Snail	29	1:1000	Rabbit	Abcam	3879S
GAPDH	37	1:3000	Rabbit	Bioworld	AP0063

### MTT assay

U87 and U251 cells after trypsin digestion were inoculated with 96-well plate (100 μL/well) (Corning, Corning, NT, USA, #3599) with a density of 2000 cells/well overnight. Afterwards, 5 mg/mL MTT (3-(4, 5-dimethylthiazol-2-yl)-2, 5-diphenyl tetrazolium bromide) (Genview, Beijing, China; # JT343) 20 µL was added 4 h before the end of culture without changing liquid. After 4 h, the medium was completely removed, 100 μL dimethyl sulfoxide (DMSO) was added. The mixed solution was oscillated for 5 min, OD value was detected by the enzyme-connected immunodetector 490/570 nm and the data were recorded for analysis.

### Apoptotic assay

The U87 and U251 cells were cultured with 6-well plate, 2 mL/well, digested with trypsin 5 days after lentivirus transfection, complete medium was resuspended into cell suspension. Cell precipitation was washed by D-hanks precooling at 4 °C (pH = 7.2–7.4). The cells were washed with 1× binding buffer for precipitation, and centrifuged at 1300 rmp for 3 min. 1× binding buffer 200 μL was added to suspend cell precipitation, followed by 10 μL Annexin V-APC staining at room temperature and leave it in the dark for 15 min. Finally, 1× binding buffer 500 μL was added and tested with flow cytometer (Millipore, Guava easy Cyte HT) and fluorescent microscope (OLYMPUS, IX71).

### Transwell assay

The U87 and U251 cells were digested with trypsin, and the cell suspension was prepared by resuspension with low serum medium. At the same time, 100 μL serum-free medium was added and placed in the incubator for 1–2 h. Removed the medium from the small chamber and added 600 μL containing 30% FBS to the lower chamber. The cells were inoculated in 24 well plates 80,000 cells/well, 100 μL/well in inner chamber and 600 μL/well in outer chamber for 24 h. Place the chamber upside down on the blotting paper to remove the medium and gently remove the metastatic cells using a cotton swab. Added 400 μL stain to the hole in the 24-well plate and soak the chamber in the staining solution for 20 min, dyed the cells on the lower surface of the membrane to transfer the cells. Soaked the chamber in a large water cup and rinsed it in the air after washing it several times. Microscope photo membrane dissolved at 10% acetic acid, detection of absorbent OD540.

### Human apoptosis antibody array

Related protein of U87 cells in the human apoptosis signaling pathway were detected using the apoptotic antibody array kit (Abcam, USA, # ab134001). After 3 days of lentivirus transfection, the sample cells were collected, washed with PBS, and lysed at 4 °C for 30 min, gently shaken well. The total extracted protein was diluted with the array diluent buffer kit to 0.5 mg/mL. Each array antibody membrane was blocked with blocking buffer for 30 min at room temperature, which incubated at 4 °C and gently shaken overnight. HRP linked Streptavidin was added to the membranes. Protein was visualized using ChemiDoc XRS chemiluminescence detection and imaging system. The density of the spots was quantitated using Quantity One software and normalized to the *α*-tubulin levels.

### Animal xenograft model and intracranial xenograft model

Animal research was approved by the Ethics committee of Shanghai Tongji University conducted in accordance with guidelines and protocols for animal care and protection. BALB/c female nude mice (4 weeks old) were purchased from Shanghai Jiesijie Experimental Animals Co., Ltd (Shanghai, China). U87 cells with shDEPDC1B or shCtrl were subcutaneously injected into BALB female nude mice (4 × 10^6^ cell per mouse). Data were collected (the weight and volume of the tumor) after 15 days of injection of U87 cells, and then measured per week for 27 days. 10 min before in vivo imaging, anesthesia was performed by inhaling with 3% isoflurane. Subsequently, the tumor load was evaluated weekly with bioluminescence imaging, and the IVIS spectral imaging system (emission wavelength 510 nm) analyzed.

Firstly, U87 cells with shDEPDC1B or shCtrl were injected under the nude mouse skin to form a subcutaneous transplant tumor, and then the subcutaneous transplant tumor tissue was drilled into the nude mouse skull quantitatively to make a model of the nude intracerebral transplant tumor. Next, survival status of nude mice after transplantation was observed. After 27 days, the naked mouse brain was dissected and sectioned. Finally, the morphology of orthotopic transplantation tumor was observed for biofluorescence imaging. After that, the mice were sacrificed by injection of sodium pentobarbital, and the tumors were removed and collected. Finally, the tumor was weighed and photographed.

### Ki67 staining

Tumor tissues were obtained from sacrificial mice and intracranial, and then made into tissue sections. After repairing and blocking with the citrate antigen, antibody Ki67 (1: 200, Abcam, USA, # ab16667) was added to the shDEPDC1B or shCtrl, respectively. Subsequently, mixed and incubated overnight at 4 °C. PBS elution for several times, IgG (1:400, Abcam, USA, # ab6721), secondary antibody was added and incubated at room temperature for 30 min, PBS was washed again. Tissue slices were first stained with DAB, and then with hematoxylin. Images were collected with a photomicroscope and analyzed.

### Statistical analysis

The data are expressed as mean ± SD (n ≥ 3) and analyzed using GraphPad Prism 6 software (GraphPad Software Inc., San Diego, CA, USA). qRT-PCR was analyzed by 2^−∆∆CT^ method. T-test were used to compare the difference. P values less than 0.05 were considered statistically significant.

## Results

### DEPDC1B is upregulated in GBM patients

**Fig. 1:**
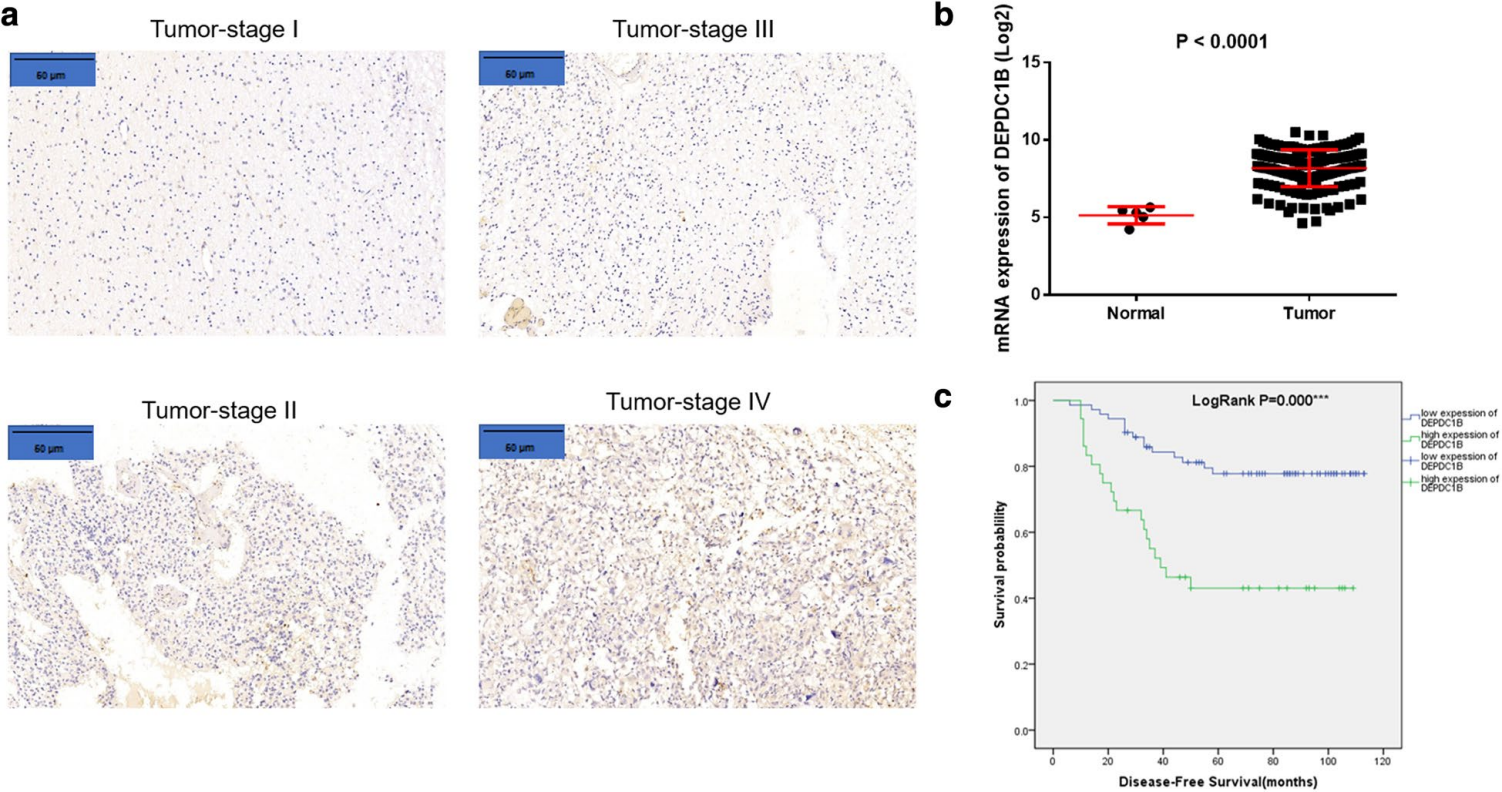
DEPDC1B is highly expressed in GBM tissues. **a** The expression of DEPDC1B in the tumor tissues detected by IHC (scale bar = 60 μm). **b** The mRNA expression of DEPDC1B in the normal and tumor samples in the TCGA-GBM sample database. **c** Kaplan–Meier survival analysis DEPDC1B expression and overall survival of GBM. The data were expressed as the mean ± SD (n = 3), *P < 0.05, **P < 0.01, ***P < 0.001

First, results of IHC shown that the expression of DEPDC1B in the tumor tissues was significantly higher than that in the normal tissues (Fig. 1a). Moreover, we got TCGA data from the website https://portal.gdc.cancer.gov/, which includes 108 GBM tissues and matched normal tissues. And the results were consistent with the data of IHC (Fig. 1b) (P < 0.0001). Based on Mann–Whitney U analysis (Table 1), we found that the significant correlation between DEPDC1B expression and pathological grading as well as tumor recurrence. Results of Spearman correlation analysis (Table 2) also verified that the Mann–Whitney U analysis. In a word, the expression of DEPDC1B increased with the progression of tumor malignancy, which was also positively associated with high risk of tumor recurrence through the Kaplan–Meier survival analysis (Fig. 1c). Comprehensive analysis affirmed that DEPDC1B may be related to the development and prognosis of GBM.

**Table 1:** Relationship between DEPDC1B expression and tumor characteristics in patients with glioma

Features	No. of patients	DEPDC1B expression		P value
		Low	High	
All patients	108	72	36	
Age (years)				0.216
≤ 41	45	33	12	
> 41	63	39	24	
Gender				0.560
Male	74	48	26	
Female	34	24	10	
Tumor recurrence				0.002
No	53	43	10	
Yes	55	29	26	
Grade				0.000
I	13	13	0	
II	52	47	5	
III	29	9	20	
IV	14	3	11

**Table 2:** Relationship between DEPDC1B expression and tumor characteristics in patients with glioma

	DEPDC1B
Grade	
Pearson correlation	0.645
Significance (double tail)	0.000
N	108
Tumor recurrence	
Pearson Correlation	0.301
Significance (double tail)	0.002
N	108

### DEPDC1B is downregulated in shRNA mediated knockdown of U87 and U251 cells

**Fig. 2:**
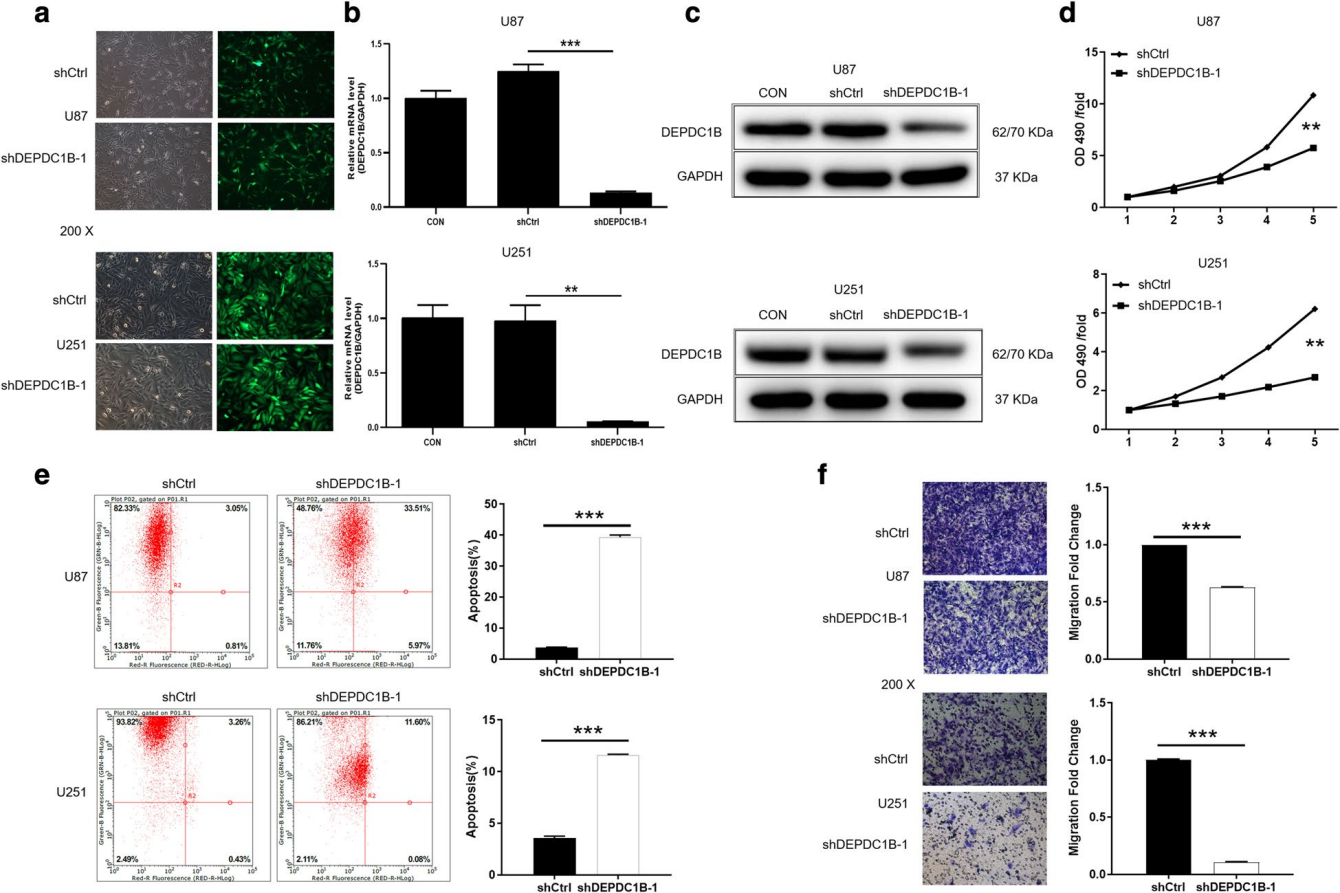
Comparison of proliferation, apoptosis and migration of GBM cells in ShDEPDC1B-1 and shCtrl groups. **a** Transfection efficiency for U87 and U251 cells was evaluated by expression of green fluorescent protein 72 h post-infection. **b**, **c** The specificity and validity of the lentivirus-mediated shRNA knockdown of DEPDC1B expression was verified by qRT-PCR (**b**) and western blot analysis (**c**). **d** Cell proliferation of U87 and U251 cells with or without knockdown of DEPDC1B was evaluated by MTT assay. **e** Flow cytometry analysis based on Annexin V-APC staining was utilized to detect the percentage of early apoptotic cell for U87 and U251 cells with or without knockdown of DEPDC1B. The X axis indicated the cell apoptosis while the Y axis indicated the green fluorescence detected from the GFP tagged on lentivirus (shDEPDC1B-1 and shCtrl). **f** Cell migration of U87 and U251 cells with or without knockdown of DEPDC1B was evaluated by Transwell assay. The data were expressed as mean ± SD (n = 3), *P < 0.05, **P < 0.01, ***P < 0.001

GBM cell lines U87 and U251 were selected as cell models for subsequent experiments. The cells were transfected with shDEPDC1B-1 or shDEPDC1B-2 to silence DEPDC1B, and that transfected with shCtrl was used as negative control. Fluorescence imaging showed that the transfection efficiency of U87 and U251 were both > 80% (Fig. 2a). Moreover, results of qRT-PCR displayed that the knockdown efficiencies of DEPDC1B-1 in U87 and U251 were 83.3% (*P *< 0.01) and 92.5% (*P* < 0.05), respectively (Fig. 2b). Similarly, western blot also displayed that expression of protein DEPDC1B-1 was downregulated in U87 and U251 cells. These data suggested that DEPDC1B-1 knockdown cell models were constructed successfully (Fig. 2c).

### Knockdown of DEPDC1B inhibited GBM cells proliferation

In order to further study the role of DEPDC1B in GBM development, MTT assay was adopted. The results of measurement for 5 days showed that the value of OD490 in shDEPDC1B-1 group was significantly lower than that in shCtrl group, suggesting that knockdown of DEPDC1B in U87 and U251 cells suppressed cell proliferation (*P *< 0.001) (Fig. 2d). The results confirmed that DEPDC1B may play a vital role of GBM on cell proliferation.

### Knockdown of DEPDC1B cells promoted GBM cells apoptosis

Subsequently, flow cytometry was used to determine the percentage of apoptotic cells among the cells transfected with shDEPDC1B-1 or shCtrl. Compared with shCtrl group, the downregulation of DEPDC1B greatly promoted the apoptosis of U87 and U251 cells. The apoptosis rates of U87 and U251 increased by 35.59% and 8.01%, respectively (*P *< 0.001) (Fig. 2e). Therefore, we can infer that knockdown of DEPDC1B enhanced cell apoptosis of GBM.

### Knockdown of DEPDC1B inhibited GBM cells migration

In addition, Transwell assay was performed to detect the ability of migration of U87 and U251 cells. In U87 cells, the migration rate of shDEPDC1B-1 group was 37% lower than that of control group (*P *< 0.001). In addition, in U251 cells, shDEPDC1B-1 group almost lost the ability of migration compared with the control group (*P *< 0.001) (Fig. 2f). Obviously, the knockdown of DEPDC1B inhibited the cells migration ability of GBM.

**Fig. 3:**
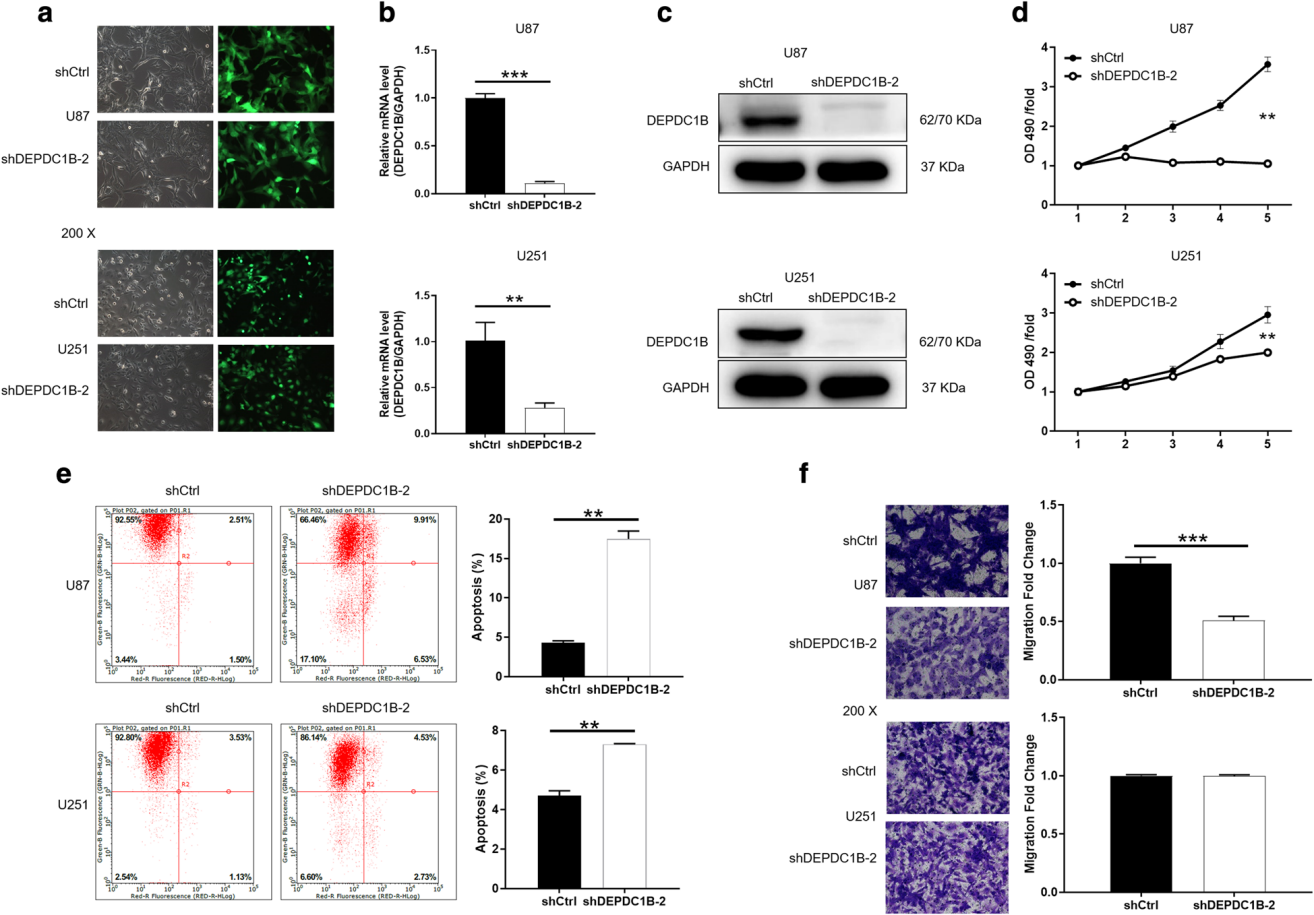
Comparison of proliferation, apoptosis and migration of GBM cells in ShDEPDC1B-2 and shCtrl groups. **a** Transfection efficiency for U87 and U251 cells was evaluated by expression of green fluorescent protein 72 h post-infection. **b**, **c** The specificity and validity of the lentivirus-mediated shRNA knockdown of DEPDC1B expression was verified by qRT-PCR (**b**) and western blot analysis (**c**). **d** Cell proliferation of U87 and U251 cells with or without knockdown of DEPDC1B was evaluated by MTT assay. **e** Flow cytometry analysis based on Annexin V-APC staining was utilized to detect the percentage of early apoptotic cell for U87 and U251 cells. The X axis indicated the cell apoptosis while the Y axis indicated the green fluorescence detected from the GFP tagged on lentivirus (shDEPDC1B-2 and shCtrl). **f** Cell migration of U87 and U251 cells with or without knockdown of DEPDC1B was evaluated by Transwell assay. The data were expressed as mean ± SD (n = 3), *P < 0.05, **P < 0.01, ***P < 0.001

In addition, we cited another new and effective shRNA to knockdown DEPDC1B (shDEPDC1B-2), and establish a cell model of downregulation of DEPDC1B (Fig. 3a–c). Similarly, we used the downregulation of DEPDC1B of U87 and U251 to carry out cell function experiments, and the results were illustrated in Fig. 3d–f. Consistently, compared with shCtrl, the group of shDEPDC1B-2 can inhibit the progression of glioblastoma by inhibiting proliferation, enhancing apoptosis and suppressing migration. Therefore, we determined that the inhibitory effect was caused by the knockdown of DEPDC1B.

### Exploration of downstream molecular mechanism of DEPDC1B in GBM cells

**Fig. 4:**
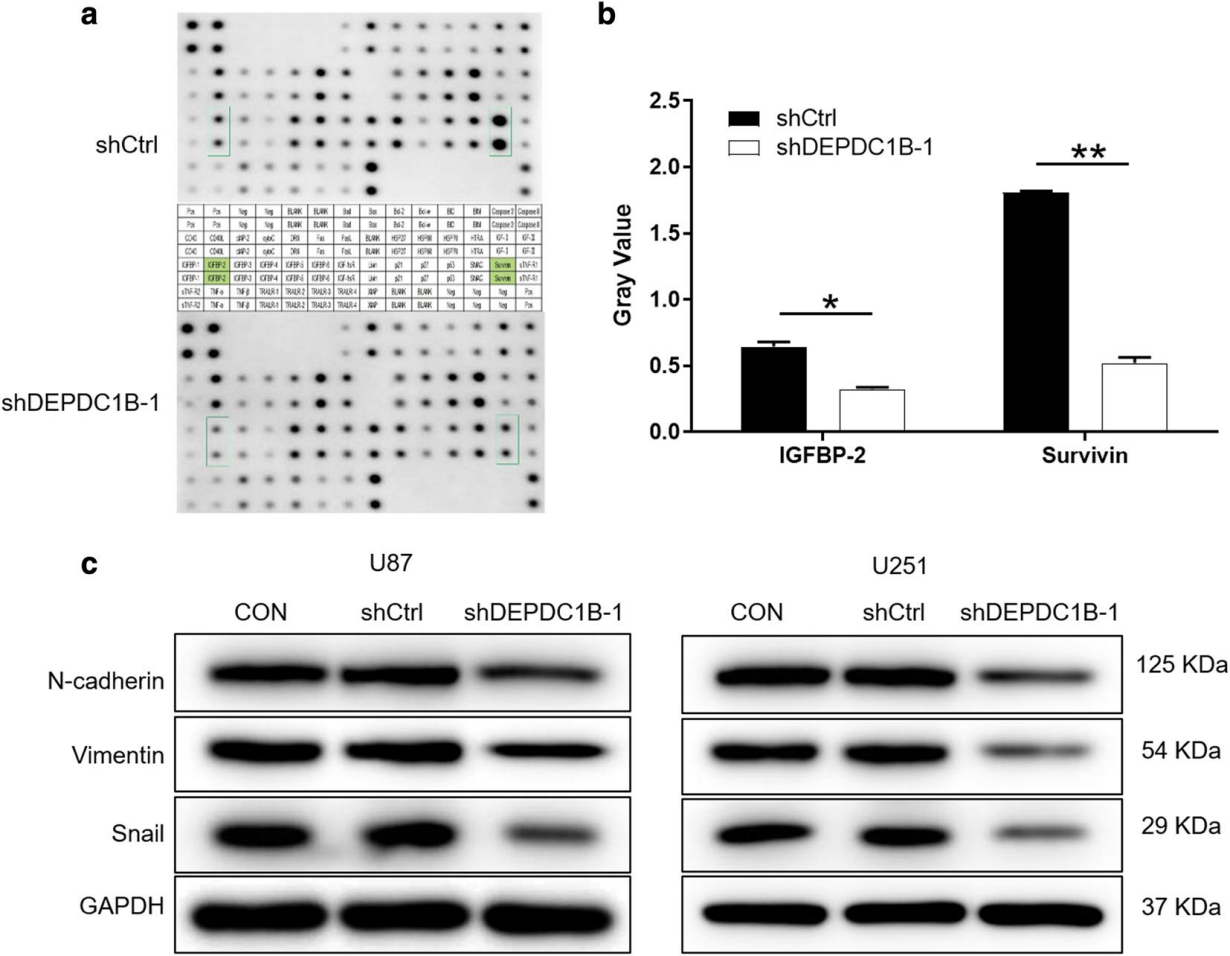
Exploration of downstream molecular mechanism of DEPDC1B in GBM cells. **a** Human apoptosis antibody array analysis was performed in U87 cells with or without DEPDC1B knockdown. **b** Densitometry analysis was performed and the gray values of differentially expressed proteins were shown. **c** The expression of epithelial-mesenchymal transition (EMT) proteins were observed by western blot in U87 and U251 cells. The data were expressed as mean ± SD (n = 3), *P < 0.05, **P < 0.01, ***P < 0.001

For exploring the potential mechanism of the regulation ability of DEPDC1B knockdown in GBM, human apoptosis antibody array was performed to analyze the differential expression of 43 proteins in U251 cells between shDEPDC1B-1 and shCtrl groups. After silencing DEPDC1B in U251 cells, the expression levels of IGFBP-2 and Survivin proteins in the human apoptosis signaling pathway were significantly downregulated (*P *< 0.05) (Fig. 4a, b). These results were consistent with the aforementioned cellular experiments especially the cell apoptosis assay. Moreover, western blot was used to detect EMT related protein expression in U87 and U251 cells. Compared with the negative group, protein expressions of N-cadherin, Vimentin and Snail were downregulated in group shDEPDC1B-1 (Fig. 4c).

### Knockdown of DEPDC1B in GBM cells impaired tumor growth in vivo

**Fig. 5:**
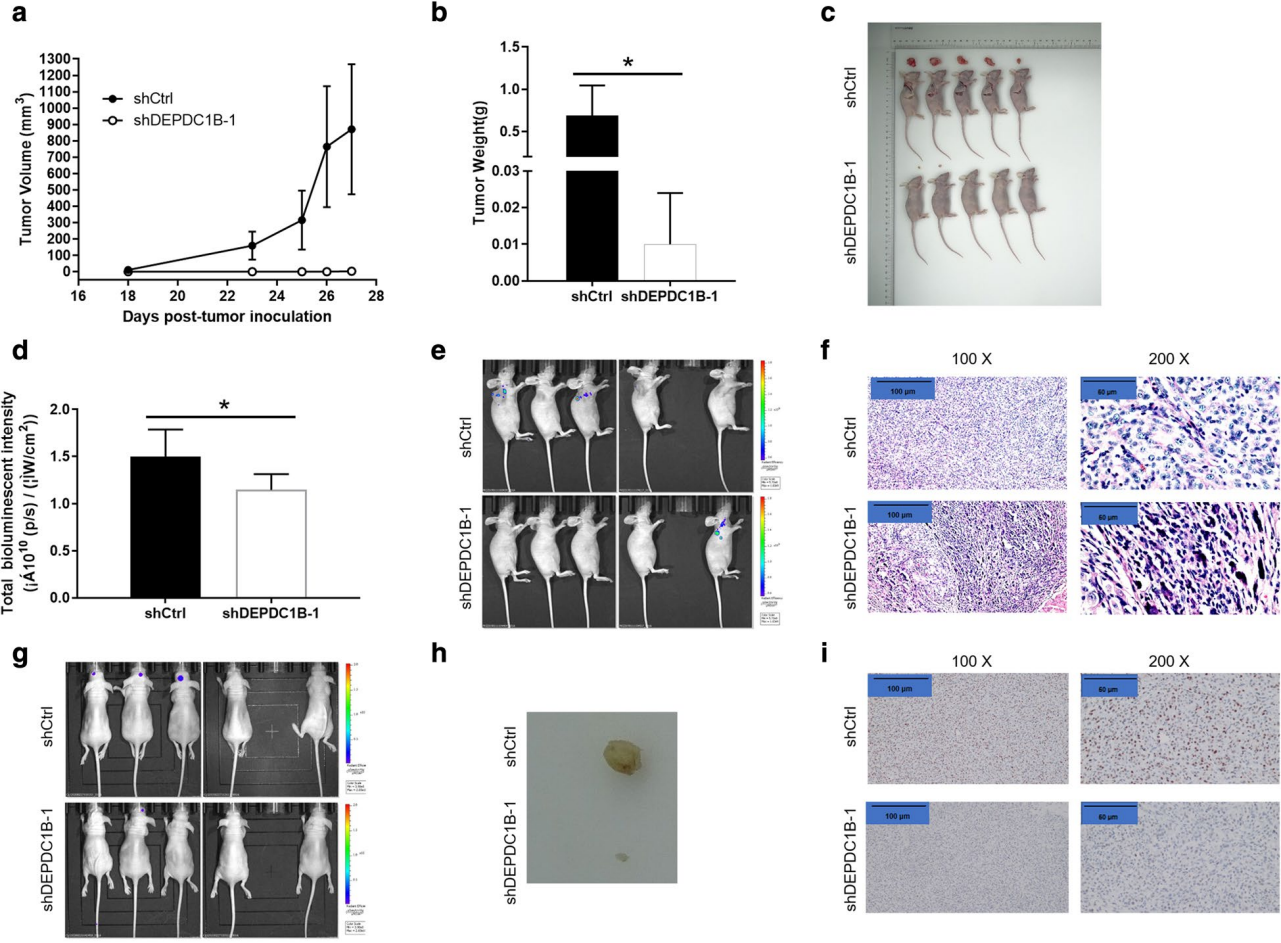
Knockdown of DEPDC1B inhibits tumor growth in mice xenograft models and intracranial xenograft models. **a** The volume of tumors in shCtrl group and shDEPDC1B-1 group was measured 18, 20, 22, 24, 26 days’ post-injection. **b** The average weight of tumors in shCtrl group and shDEPDC1B-1 group. **c** The tumors of mice in shCtrl group and shDEPDC1B-1 group. **d** The total bioluminescent intensity of tumors in shCtrl group and shDEPDC1B-1 group. **e** The bioluminescence imaging of tumors in shCtrl group and shDEPDC1B-1 group. **f** The Ki67 staining of tumor tissues in shCtrl group and shDEPDC1B-1 group (scale bar = 100 μm). **g** The bioluminescence imaging of intracranial tumors in shCtrl group and shDEPDC1B-1 group. **h** The intracranial tumors in shCtrl group and shDEPDC1B-1 group. **i** The Ki67 staining of intracranial tumor tissues in shCtrl group and shDEPDC1B-1 group (scale bar = 100 μm). The data were expressed as mean ± SD (n = 3), *P < 0.05, **P < 0.01, ***P < 0.001

The above experiments confirmed that DEPDC1B could promote cell proliferation and migration and inhibit cell apoptosis in GBM in vitro. We still wondered whether knockdown of DEPDC1B affects tumor growth in vivo. Therefore, U87 cells were subcutaneously injected into nude mice to establish xenograft model and intracranial xenograft model. The results showed that the average volume of tumor of shDEPDC1B-1 group was significantly decreased by 867.83 ± 393.13 mm^3^ than that of the shCtrl group, (P < 0.01) (Fig. 5a). The average tumor weight of mice inoculated with shDEPDC1B-1, which was markedly lower 0.682 ± 0.340 g than that of the shCtrl group (*P *< 0.05) (Fig. 5b, c). Additionally, bioluminescence imaging and IVIS Spectrum Imaging System suggested that fluorescence expression decreased in shDEPDC1B-1 group than that in shCtrl group (*P *< 0.05) (Fig. 5d, e). Moreover, Ki67 staining showed that the proliferation index of tumor tissue in shDEPDC1B-1 group was significantly slower than that in negative group (Fig. 5f). In addition, intracranial xenograft model was also constructed and the corresponding results were shown in Fig. 5g–i, further confirming the suppressed tumor growth by DEPDC1B knockdown. Taken together, these results indicated that knockdown of DEPDC1B impaired tumorigenicity in vivo, which was in accordance with the aforementioned data in vitro.

## Discussion

GBM is the most common malignant brain tumor in adults and one of the most fatal primary tumors [16]. It is characterized by poor prognosis and with an extreme low survival rate [16]. Glioma stem cells typically reside in the hypoxic core of tumors, so these tumors are highly incurable and prone to relapse [17]. This lethal malignant tumor of the central nervous system (CNS) showing dismal prognosis under the standard care of surgery, adjuvant radiotherapy and temozolomide (TMZ) [1]. Radiotherapy and TMZ chemotherapy induced DNA damage in GBM tumor cells by generating double-chain break (DSB) and single-chain break (SSB), respectively [1]. Recently, Filbin et al. demonstrated synergistic inhibition of PI3K and Hedgehog (Hh) signaling pathway together as a better therapeutic approach in GBM [18]. Biswas et al., previously reported that there is a strong possibility of induction of apoptosis cellular senescence in GBM neoplastic cells if the Hh signaling pathway is kept suppressed while treating GBM with TMZ [19]. Clinically, inhibition of this pathway could be a potential strategy to enhance sustained TMZ-response in this malignancy. Agrawal et al. identified a novel role of NeuroD2 as a tumor suppressor and prognostic biomarker in GBM the levels of which are tightly regulated by p53 and miR-210 [20]. Overexpressing NeuroD2 may potentially be a simple and efficient therapeutic strategy to inhibit the malignant phenotype of GBM cells [20]. Therefore, substantial great efforts have been made in developing new approaches for radiotherapy modalities, targeted chemotherapy and gene therapy. However, the MS for patients with newly diagnosed GBM have improved only modestly during the past 10 years. Thus, there is a crucial need to identify new therapeutic targets for treating GBM.

DEPDC1B was found to be highly expressed in placenta and testis, but less expressed in heart and small intestine [21]. Marchesi et al. described an adherence-dependent mitotic checkpoint and determined that DEPDC1B is a factor that coordinates desattachment with the ability of cells to enter mitosis [22]. Previous profiling of DEPDC1B mRNA in MDA-MB 231 breast cancer cells showed that it was associated with decreased cell death and increased cell proliferation [23]. In addition, the relationship between abnormal DEPDC1B expression and breast cancer, oral cancer, non-small cell lung cancer and other malignant tumors has been reported [21, 23, 24]. Su found that DEPDC1B plays a role in the development of oral cancer and revealed that proliferation was linked to a novel DEPDC1B–Rac1–ERK1/2 signaling axis in oral cancer cell lines [21]. Yang demonstrated that DEPDC1B was able to activate Wnt/β-catenin signaling, and that depletion of TCF4 or LEF1 abrogated the biological effects of DEPDC1B on cellular migration and invasion [24]. The role of DEPDC1B in prostate cancer was reported by Huang et al. DEPDC1B expression significantly upregulated the level of cancer tissue and its mRNA in prostate gland could be used as an independent prognostic indicator for survival rate of prostate cancer patients [25]. In addition, Kikuchi et al. found that inhibition of endogenous DEPDC1 expression by small interfering RNA (siRNA) inhibits GBM cell viability and induces apoptosis through NF-κB signaling [26]. Therefore, we set out to explore the effects of DEPDC1B in GBM.

To the best of our knowledge, this study is the first to investigate the relationship between DEPDC1B levels and characteristics in GBM patients. First, MTT assay, colony formation assay and flow cytometry demonstrated that DEPDC1B could promote cell proliferation and inhibit cell apoptosis in GBM. Moreover, the fact that DEPDC1B may be related to cell migration in GBM was proved by Transwell assay. In addition, in vivo studies demonstrated the decreased tumorigenicity after silencing of DEPDC1B, which is consistent with in vitro studies. Subsequently, human apoptosis signaling pathway and western blot indicated that IGFBP-2, Survivin and N-cadherin, Vimentin, Snail were downregulated with the DEPDC1B knock down. Our study confirmed that DEPDC1B expression is significantly associated with overall survival for GBM. These findings highlighted the significance of DEPDC1B in tumor and implicate DEPDC1B as a promising candidate target for GBM treatment.

In the same way, studies over past decades have provided molecular information on GBM, such as EMT. EMT is a developmental plasticity process involving a loss of epithelial features, like expression of the cell junction molecule E-cadherin, and a gain in mesenchymal features [27]. For example, Plasma IGFBP-2 levels were significantly correlated with tumor volume and independently associated with poor overall survival in patients with GBM [28]. Abdolhoseinpour et al. found that Serum and tissue expression levels of IGFBP-2 and IGFBP-3 can be used as potential biomarkers to predict the progression and survival of GBM [29]. It is concluded that Survivin high expression, singular vascular morphology and secondary GBM are related to low survival rate, while microvascular density is not related to survival rate [30]. Nuclear expression of Survivin is a factor for a poor prognosis in GBM patients. Subcellular localization of Survivin can help to predict OS in GBM patients treated with the standard protocol [31]. In particular, we found that in GBM cells, when expression of DEPDC1B is silenced, EMT protein such as N-cadherin, Snail and Vimentin were also downregulation, indicating that DEPDC1B regulate EMT. Matrix metalloproteinases (MMPs) and their related family “a disintegration proteins and metalloproteinases” (ADAM) can both promote cell invasion, and their substrate N-cadherin is involved in the proliferation and metastasis of GBM [32]. Zhao et al. concluded that high expression of Vimentin is associated with progression and a poor outcome in glioblastoma [33]. In addition, Snail expression is regulated by activation of STAT3 because of the high level of phosphorylation of STAT3 in recurrent GBM tumors demonstrated by Liang et al. [34]. In conclusion, the results of these studies are consistent with the results of this paper. Therefore, it was speculated that DEPDC1B knockdown promoted the apoptosis and inhibited the migration activity of GBM cells by regulating EMT and apoptotic proteins. Although we have provided valid evidence of DEPDC1B role in GBM, there are still imperfections in this study. For example, the number of specimens included in this study is limited, and the potential mechanism of DEPDC1B-mediated GBM regulation is unclear. In order to conquer GBM, these issues still need to be explored.

## Data Availability

The datasets used and/or analyzed during the current study are available from the corresponding author on reasonable request.
